# Changes in remote myocardial tissue after acute myocardial infarction and its relation to cardiac remodeling: A CMR T1 mapping study

**DOI:** 10.1371/journal.pone.0180115

**Published:** 2017-06-23

**Authors:** P. Stefan Biesbroek, Raquel P. Amier, Paul. F. A. Teunissen, Mark B. M. Hofman, Lourens F. H. J. Robbers, Peter M. van de Ven, Aernout M. Beek, Albert C. van Rossum, Niels van Royen, Robin Nijveldt

**Affiliations:** 1 Departments of Cardiology, VU University Medical Center, Amsterdam, the Netherlands; 2 Netherlands Heart Institute, Utrecht, the Netherlands; 3 Physics & Medical Technology, VU University Medical Center, Amsterdam, the Netherlands; 4 Epidemiology & Biostatistics, VU University Medical Center, Amsterdam, the Netherlands; Indiana University School of Medicine, UNITED STATES

## Abstract

**Objectives:**

To characterize the temporal alterations in native T1 and extracellular volume (ECV) of remote myocardium after acute myocardial infarction (AMI), and to explore their relation to left ventricular (LV) remodeling.

**Methods:**

Forty-two patients with AMI successfully treated with primary PCI underwent cardiovascular magnetic resonance after 4–6 days and 3 months. Cine imaging, late gadolinium enhancement, and T1-mapping (MOLLI) was performed at 1.5T. T1 values were measured in the myocardial tissue opposite of the infarct area. Myocardial ECV was calculated from native- and post-contrast T1 values in 35 patients, using a correction for synthetic hematocrit.

**Results:**

Native T1 of remote myocardium significantly decreased between baseline and follow-up (1002 ± 39 to 985 ± 30ms, p<0.01). High remote native T1 at baseline was independently associated with a high C-reactive protein level (standardized Beta 0.32, p = 0.04) and the presence of microvascular injury (standardized Beta 0.34, p = 0.03). ECV of remote myocardium significantly decreased over time in patients with no LV dilatation (29 ± 3.8 to 27 ± 2.3%, p<0.01). In patients with LV dilatation, remote ECV remained similar over time, and was significantly higher at follow-up compared to patients without LV dilatation (30 ± 2.0 versus 27 ± 2.3%, p = 0.03).

**Conclusions:**

In reperfused first-time AMI patients, native T1 of remote myocardium decreased from baseline to follow-up. ECV of remote myocardium decreased over time in patients with no LV dilatation, but remained elevated at follow-up in those who developed LV dilatation. Findings from this study may add to an increased understanding of the pathophysiological mechanisms of cardiac remodeling after AMI.

## Introduction

Following acute myocardial infarction (AMI), the infarcted myocardium undergoes a sequence of pathophysiological changes including myocardial necrosis, myocardial edema, microvascular injury, and subsequent healing with scar tissue formation [1]. There is increasing evidence indicating that also remote myocardium is subjected to pathophysiological changes after MI, although their severity and significance is still largely unknown [2, 3].

In vivo, pathological changes in myocardial tissue composition can be evaluated using cardiovascular magnetic resonance (CMR) imaging. In the past few years, several technological advances have occurred that improved the capability of CMR to characterize the myocardium. Native (i.e. pre-contrast) T1 mapping now permits direct quantification of the absolute T1 relaxation times, and can therefore be used to detect and quantify myocardial edema, without the need for a reference region [4–6]. Furthermore, post-contrast T1 mapping enables quantification of gadolinium (Gd)-based contrast accumulation within the interstitial space of the myocardium, and thereby provides a direct measure of the size of the extracellular volume (ECV) [7].

Findings from recent studies using T1 mapping suggest that tissue changes may also occur in remote myocardium after AMI, and that these changes are associated with adverse cardiac remodeling [8–12]. However, current data on native T1 and ECV of remote myocardium in AMI patients and their predictive role on left ventricular (LV) remodeling are conflicting. In one study [11], native T1 of remote myocardium decreased from baseline to 5 months after AMI, while in another study remote native T1 values remained constant [12]. Furthermore, a different study found remote native T1 to be an independent predictor of adverse LV remodeling after AMI [10], which was not found in another [11]. A better knowledge of the tissue changes in remote myocardium and their relation to adverse cardiac remodeling may help to better understand the pathophysiological mechanisms responsible for adverse cardiac remodeling after AMI, which in turn could help to identify patients at increased risk and also potential targets for therapy. The objective of this study was therefore to assess the temporal change in tissue composition of remote myocardium after AMI by performing native and post-contrast T1 mapping in both the acute phase and at 3 months of follow‐up. Second, this study was conducted to explore the relation of remote tissue alterations, as reflected by native T1 and ECV values, and LV remodeling after AMI.

## Materials and methods

### Patient population

The study was conducted in accordance with the Declaration of Helsinki and, approved by the local Medical Ethics Review Committee (VU University Medical Center), and all patients gave written informed consent [13]. Patients were selected from the PREDICT-MVI study of which the design and main results have been published previously [14]. Briefly, a total of sixty patients with acute ST-segment elevation MI successfully treated with primary PCI were prospectively included between December 2011 and February 2013. Exclusion criteria were triple vessel disease, previous coronary revascularization procedures, previous MI, unstable hemodynamics, and refusal or inability to give informed consent.

A 12-lead ECG was acquired before reperfusion and 1 hour afterwards. Absence of ST-segment resolution (STR) was defined as <30% resolution of ST-segment elevation from before to after reperfusion. Venous blood samples were collected 4 days following hospitalization. For each sample, high sensitive C-reactive protein (CRP) concentration was measured on a Cobas 6000 analyzer (Roche Diagnostics) according to the manufacturer’s instructions, and fibrinogen antigen was measured by enzyme-linked immunosorbent assay (ELISA) using antibodies from Kordia B.V. (Leiden, The Netherlands).

### CMR imaging protocol

CMR was performed on a 1.5T clinical MR system (Avanto, Siemens, Erlangen, Germany). All patients underwent CMR imaging 4–6 days and 3 months after AMI. Considering that the greatest changes in LV volume, mass and function are observed during the first few months after AMI [15], and the risk of new myocardial infarction increases with longer follow-up duration [16], the follow-up was performed at 3 months, consistent with previous studies that used CMR to assess LV remodeling after AMI [17, 18]. Cardiac function was assessed using retrospective-triggered, balanced steady-state free precession cine imaging in four-, three-, and two- chamber long-axis and short-axis orientations (typical parameters: cine imaging: voxel size ~1.6x1.9x5.0 mm, slice gap 5.0 mm, TR/TE 3.2/1.6 ms, flip angle 75°, temporal resolution <50 ms). A segmented T2 weighted turbo spin echo (T2w) sequence with fat suppression was performed in a short-axis orientation with full LV coverage. Based on abnormalities in cine wall motion and edema on T2w images, three contiguous short-axis T1 maps were acquired through the infarct core. T1 mapping was performed using a single breath-hold Modified Look-Locker Inversion Recovery (MOLLI) pulse sequence as previously described [19]. Eleven single-shot T1-weighted SSFP images were obtained with various inversion delays with a 3-3-5 scheme within 17 heartbeats (typical parameters: single breath-hold, voxel size 2.1x2.1x8 mm, field-of-view 360–400 mm, time of repetition 2.2 ms, echo time 1.1 ms) [20]. Late gadolinium enhancement (LGE) images were acquired 10–15 minutes after intravenous administration of a bolus of 0.2 mmol/kg Gd-DOTA (Guerbet, Villepinte, France), using a segmented inversion-recovery gradient-echo pulse sequence with continuous adjustment of inversion time to null normal myocardial signal (typical parameters: voxel size ~1.4x1.4x5.0mm, slice gap 5.0 mm, TR 2x RR interval, typical inversion time 250–400 ms, phase sensitive reconstruction). T1 mapping was repeated using identical slice locations as the native T1 maps at approximately 25 minutes after contrast injection for post-contrast analyses.

### CMR data analysis and definitions

All CMR images were analyzed offline using dedicated software (QMass v7.6, Medis, Leiden, the Netherlands). Cine images were manually traced for the measurement of LV end-diastolic volume (LVEDV), LV end-systolic volume (LVESV), LV mass, and to calculate LV ejection fraction (LVEF) [21]. Myocardial infarct size was quantified using the LGE images applying the full-width at half-maximum algorithm, and is expressed in percentage of LV mass [22]. Patients were categorized into large- and small MI groups, with large MI defined as > and small as < of the median of the total group. Microvascular injury (MVI) was identified on LGE images as hypo-enhanced regions within the hyper-enhanced infarcted myocardium. All volumetric, functional and LGE analysis were performed by an experienced level III CMR reader (L.R.), blinded to the T1 mapping results.

Significant LV dilatation was defined as an increase in LV end-diastolic volume of ≥15% 3 months after AMI [23].

**Fig 1:**
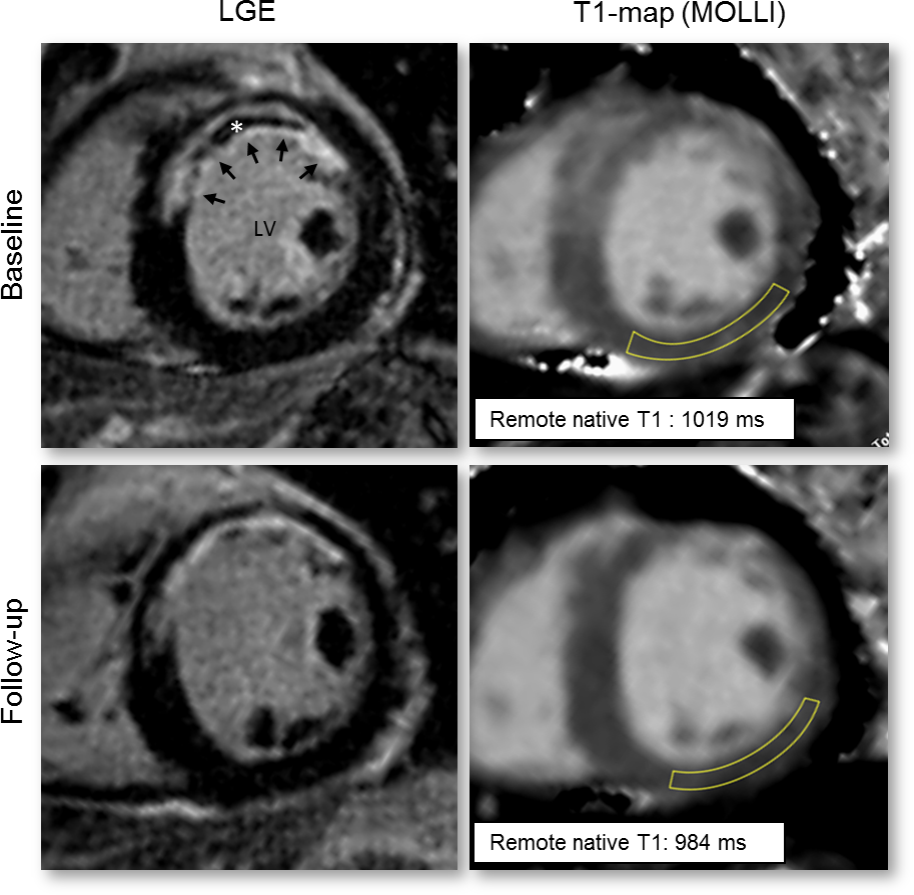
Typical location of the regions analyzed for T1 assessment. Typical location of the regions analyzed for T1 assessment in a patient with an acute anterior myocardial infarction (arrows) with microvascular obstruction (asterisk). Native T1 maps acquired at baseline (top row) and follow-up (bottom row) were matched, and specific care was taken to ensure that similar myocardial ROIs were drawn for both studies. LV = left ventricle.

Analysis of the T1 maps were preceded by examining the eleven raw T1-weighted SSFP images for the presence of off-resonance artifacts, diaphragmatic movement and variation in cardiac phase. Myocardial slices or areas with artifacts were excluded for T1 measures. For T1 measurements, infarct zone was defined as myocardial tissue with hyperenhancement on the LGE images. Remote zone was defined as myocardial tissue opposite of this infarct zone, with no visual evidence of hyper-enhancement. Fig 1 demonstrates the typical size and location of the remote zone analyzed for T1 assessment in a patient scanned at 4 days (baseline) and at 94 days (follow-up) after AMI. Regions of interest (ROI) were manually delineated within remote myocardium on automatically generated grayscale T1 maps (native and post-contrast) by consensus of two experienced CMR readers (P.S.B. and R.N.). Specific care was taken to ensure that the myocardial ROIs were taken within the myocardium to avoid partial volume effects, and to ensure adequate margins of separation between infarct zone and remote zone. For blood T1 measurement, a ROI was placed in the center of the LV cavity excluding papillary muscles. T1 relaxation times were calculated as the average of the values of the 3 short-axis slices. Native T1 values were normalized to the heart rate during CMR data acquisition, using a simulation of the MOLLI sequence as was described and validated [24]. Finally, synthetic ECV was calculated from native and post-contrast T1 measurements using a correction for synthetic hematocrit, according to a method previously described [25].

### Statistical analysis

Continuous variables are presented as mean with standard deviation (±) for normally distributed variables, median and interquartile range (IQR) for non-normally distributed variables, and as frequencies and percentages for categorical data. Histograms were used to determine if continuous variables were normally distributed. CRP was log transformed to obtain a normal distribution. Mean values were compared between subgroups of patients using an independent-samples t-test with Welch’s correction when appropriate. Means of baseline and follow-up measurements were compared using a paired t-test. Univariate and multivariate linear regression analyses were used to identify the baseline variables that were associated with dependent variables including remote native T1, and the change in LVESV and LVEDV between baseline and follow-up at 3 months. Variables that were statistically significant in univariate analyses (p<0.05) were subsequently included in a multivariate regression using forward selection. The strict criterion for inclusion in multivariate analysis, being p < 0.05, was chosen in order to adhere to the rule of thumb of at least 10 observations per variable included in the model. All statistical analyses were performed using SPSS statistics (IBM SPSS Statistics 20, Chicago, IL, USA). A p value of < 0.05 was considered statistically significant.

## Results

Forty-two of the 60 AMI patients underwent a complete CMR exam 4–6 days and 3 months after primary PCI. Baseline CMR scanning was not performed in 8 patients due to technical problems (n = 1), claustrophobia (n = 4), obesity (n = 2), and PCI procedure complicated by proximal dissection (n = 1). Additionally, 6 patients refused repeat MRI and another 4 patients had an incomplete CMR exam without T1 mapping. Post-contrast T1 maps were available for 35 subjects. From the 504 T1 maps, 443 (88%) were eligible for analysis. Clinical demographics and procedural characteristics of the 42 AMI patients with complete paired cine, LGE and native T1 mapping images are shown in Table 1.

**Table 1:** Patient characteristics.

No. of patients	42
Age (yrs)	60±9
Body mass index (kg/m^2^)	26±3
Male sex (n)	34 (81)
Risk factors (n)	
Diabetes	2 (5)
Hypertension	7 (17)
Hypercholesterolemia	6 (14)
Current or history of smoking	34 (81)
Family history of CVD	19 (45)
Time from symptom onset to reperfusion (min)	147 ± 67
Culprit artery (n)	
RCA	14 (33)
LAD	24 (57)
LCX	4 (10)
Single-vessel disease	28 (67)
ST-segment resolution post PCI (n)	
Complete, ≥70%	20 (48)
Incomplete, 30% to <70%	16 (38)
None, ≤30%	6 (14)
High sensitive C-reactive protein (mg/L)	9.7 (5.0–19.1)
CMR parameters at 4–6 days	
LV end-diastolic volume (mL)	183±35
LV end-systolic volume (mL)	91±28
LV ejection fraction (%)	51±8
Infarct size (proportion of LV mass) (%)	16 (8–26)
MVI present (n)	23 (55)
CMR parameters at 3 months	
LV end-diastolic volume (mL)	189±45
LV end-systolic volume (mL)	95±39
LV ejection fraction (%)	52±10
Infarct size (proportion of LV mass) (%)	12 (7–19)

CVD = cardiovascular disease; PCI = percutaneous coronary intervention; RCA = right coronary artery; LAD = left anterior descendens; MR = magnetic resonance; LV = left ventricle; MVI = microvascular injury; min = minutes; ml = milliliters.

### Baseline values and changes over time in remote T1 and ECV

CMR exams were performed at a mean of 4 ± 1 days and 96 ± 11 days after AMI. In the total cohort of 42 patients, median LGE infarct size at baseline was 16% (IQR: 8–26%), and LGE defined MVI was present in 55% of the patients. The 35 patients with both native- and post-contrast T1 mapping performed and the 8 patients with only native T1 mapping performed had comparable infarct sizes (median 16, IQR 8–27 vs. median 14, IQR 5–25%, p = 0.87) and similar rates of MVI (54% vs. 57%, p = 0.89).

**Fig 2:**
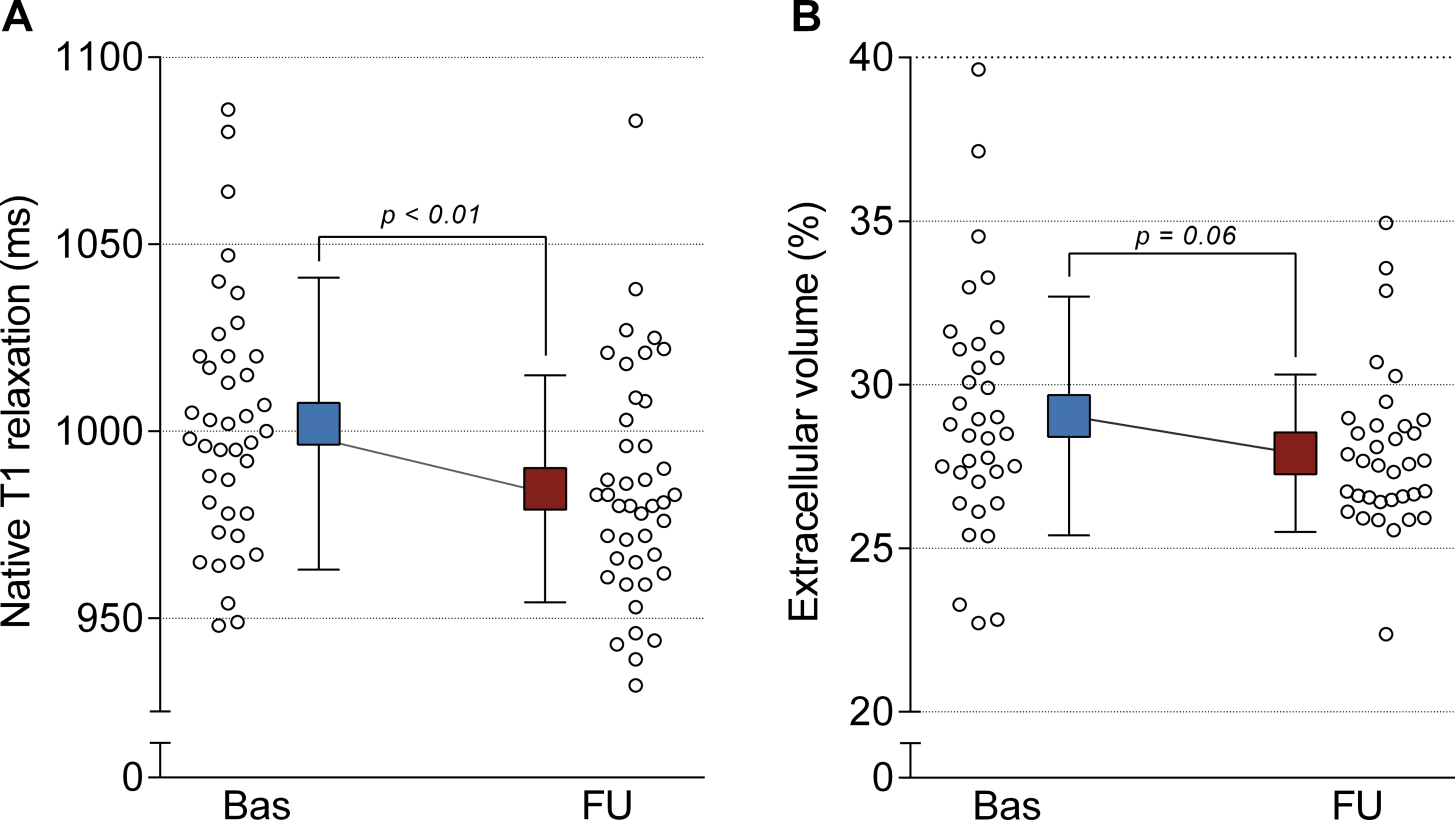
**Native T1 relaxation times (A) and extracellular volume (B) of remote myocardium 4–6 days and 3 months after myocardial infarction.** Box and error bars represent means and standard deviations.

At baseline, average native T1 of remote myocardium was 1002 ± 39ms. Three months after the MI, native T1 values of remote myocardium showed a minor but significant decrease to 985 ± 30ms, p<0.01 (Fig 2), suggestive of resolution of edema after the acute phase. A significant decrease in native T1 was also found for the uncorrected values (963 ± 34 to 950 ± 27ms, p<0.01). In the total group, the ECV of remote myocardium did not show a statistically significant change over time (29 ± 3.6% to 28 ± 2.4%, p = 0.06)(Fig 2), which could be due to opposite effects of edema resolution and fibrosis formation on ECV. There were no significant differences between patients with single- and double-vessel disease in terms of remote native T1 or ECV.

### Remote T1 and ECV in relation to AMI severity and inflammatory markers

**Fig 3:**
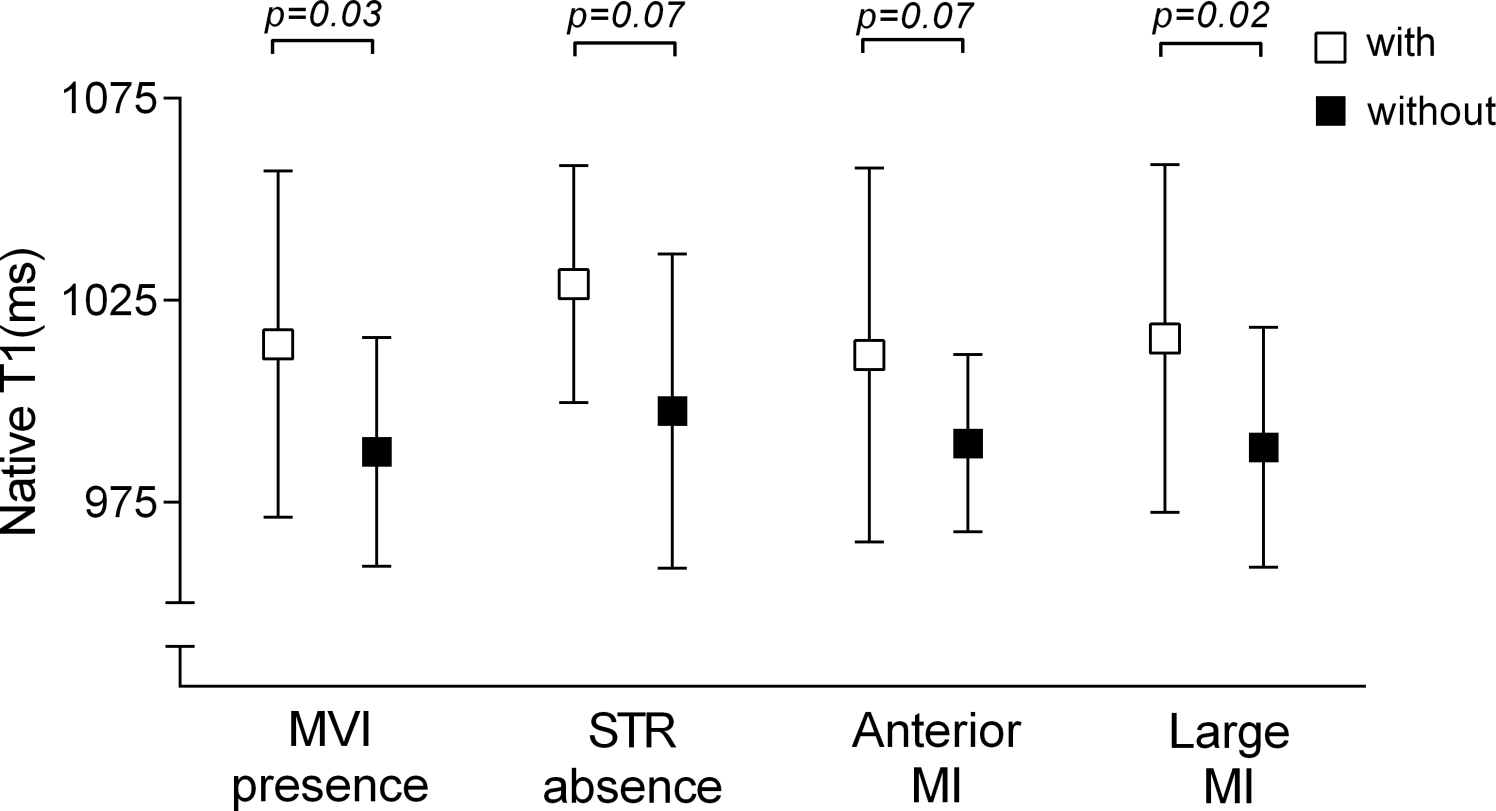
Native T1 values of remote zone myocardium between subgroups. Native T1 values of remote zone myocardium between patients with (open squares) and without (filled squares) microvascular injury (MVI), anterior myocardial infarction (MI), absence of ST-segment resolution (STR), and large myocardial infarction (MI). Squares and error bars represent means and standard deviations.

Native T1 values of remote myocardium were significantly higher in patients with MVI (MVI: 1014 ± 43ms vs. no MVI: 987 ± 28ms, p = 0.03) and in patients with a large MI size at baseline (large MI: 1016 ± 43ms vs. small MI: 989 ± 30ms, p = 0.02)(Fig 3). Remote native T1 values were also higher in patients with an anterior MI (anterior MI: 1011 ± 46ms vs. no anterior MI: 990 ± 22ms, p = 0.07) and in patients without STR (no STR: 1029 ± 29ms vs. STR: 998 ± 39ms, p = 0.07), although the difference was not statistically significant. A multivariate regression analyses with forward selection showed presence of MVI to be independently associated with remote zone native T1. No association between infarct size and remote native T1 was found when corrected for presence of MVI (Table 2). For remote ECV, no statistically significant differences were observed between patients with and without MVI (MVI: 29 ± 4.1% vs. no MVI: 29 ± 3.1%, p = 0.54), large MI (large MI: 30 ± 4.1% vs. small MI: 28 ± 3.0%, p = 0.20), anterior MI (anterior MI: 30 ± 4.4% vs. no anterior MI: 28 ± 2.4%, p = 0.27), or STR (no STR: 32 ± 3.5%vs. STR: 29 ± 3.5%, p = 0.06).

**Table 2:** Association between remote zone native T1 and patient characteristics.

	Univariate analysis			Multivariate analysis		
	B (95% CI)	Standardized Beta	p-value	B (95% CI)	Standardized Beta	p-value
Infarct size (%)	0.97 (0.01 to 1.93)	0.31	0.047	-		
Presence of MVI	26.67 (3.43 to 49.90)	0.34	0.026	26.67 (3.43 to 49.90)	0.34	0.026
Anterior MI	21.82 (-2.07 to 45.71)	0.28	0.072			
No (≤30%) ST-segment resolution post PCI	31.47 (-2.26 to 65.20)	0.29	0.067			

B = coefficient of regression; MVI = microvascular injury; MI = myocardial infarction; PCI = percutaneous coronary intervention

Median plasma level of CRP measured 4 days after AMI was 9.7mg/L (5.0–19.1mg/L). CRP levels were significantly correlated with both remote native T1 (R: 0.40, p = 0.01) and remote ECV (R: 0.41, p = 0.02). These associations remained significant after correcting for the presence of MVI and infarct size (native T1: standardized Beta 0.32, p = 0.04; ECV: standardized Beta: 0.41, p = 0.02). Mean plasma level of fibrinogen measured 4 days after AMI was 4.1±0.9 g/L. Plasma fibrinogen was significantly associated with remote ECV (R:0.35, p = 0.045), and showed a trend towards a significant correlation with remote native T1 (R:0.26, p = 0.095).

### Remote T1 and ECV in relation to LV remodeling

**Fig 4:**
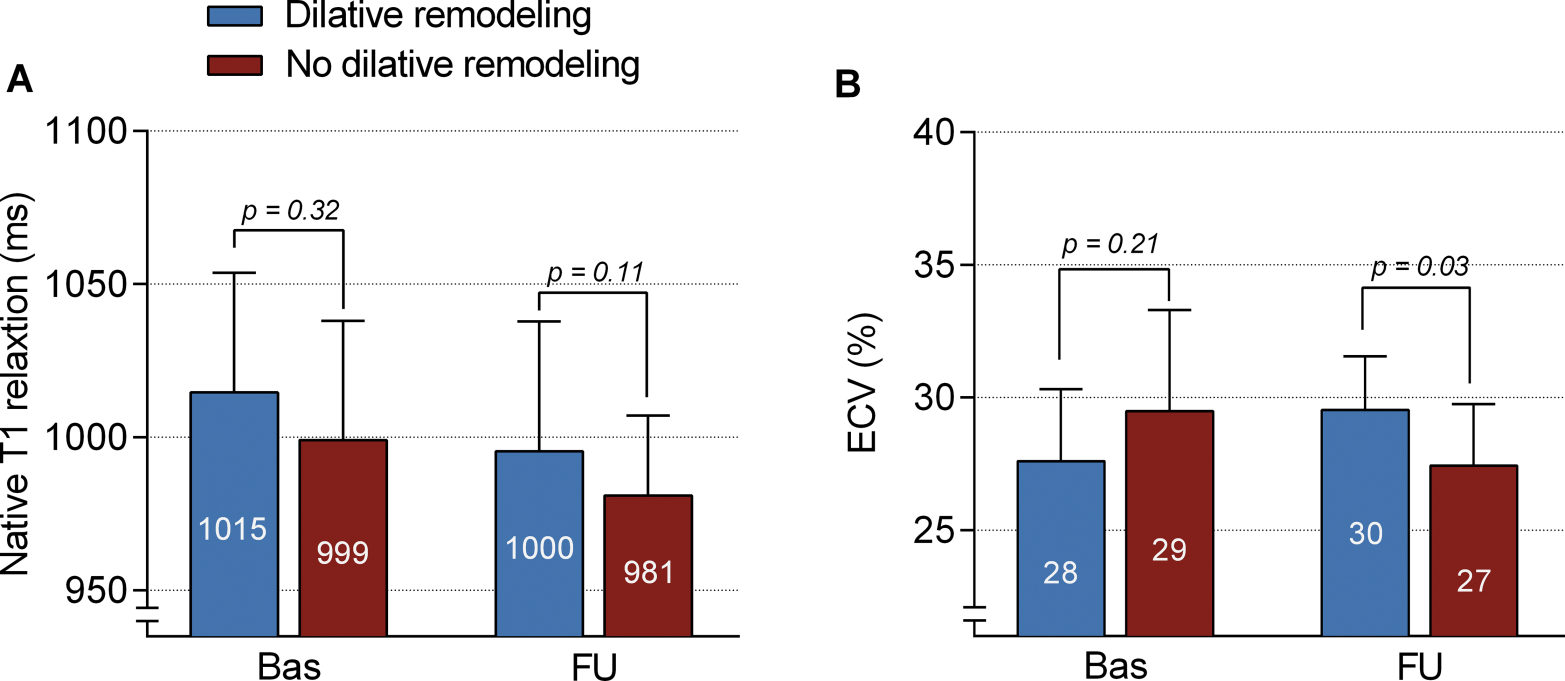
Temporal changes in remote native T1 and ECV in relation to LV dilatation. Differences at baseline and at follow-up in native T1 and ECV values of remote zone myocardium between patients with (blue bars) and without (red bars) dilative remodeling. Bars and error bars represent means and standard deviations.

Significant LV dilatation occurred in 8 patients (19%). Remote native T1 values, both at baseline and at follow-up, did not significantly differ between patients with and without significant LV dilatation (Fig 4). Furthermore, remote native T1 values at baseline showed no significant associations with changes over time in LVEDV (R: 0.16, p = 0.32) and LVESV (R: 0.17, p = 0.28). For remote ECV, values at baseline were comparable between patients with and without LV dilatation, but were significantly higher at follow-up in patients who showed LV dilation (Fig 4). Interestingly, remote ECV values significantly decreased in patients without LV dilatation (p<0.01), while they remained similar in patient with LV dilatation (p = 0.12)(Fig 4). Remote ECV values at baseline showed no significant associations with changes over time in LVEDV (R: -0.05, p = 0.79) and LVESV (R: -0.5, p = 0.76). Remote native T1 and ECV values at follow-up were significantly correlated to 3-month measurements of LGE infarct size (respectively R:0.48, p<0.01 and R:0.35, p = 0.04).

## Discussion

This study aimed to characterize the temporal alterations in native T1 and ECV of remote myocardium after AMI, and to explore their relation to LV remodeling. The main findings of the present study were as follows: *(a)* native T1 values of remote myocardium decreased from baseline to 3 months follow-up, most likely reflecting resolution of inflammatory edema, (*b*) remote native T1 values at baseline were associated with the severity of myocardial damage after AMI and CRP concentration, (*c*) ECV of remote myocardium did not show a significant change over time in the total group, but values significantly decreased in patients without LV dilatation, and (d) remote ECV values were significantly higher at follow-up in patients who developed LV dilatation.

### Changes in remote native T1 and ECV after AMI

In the present study, T1 mapping of remote myocardium was performed 4–6 days and 3 months after AMI. During this period, a slight but significant decrease in remote native T1 was observed. Our findings are consistent with a recent study by Bulluck et al. who have described a reduction in native T1 values of remote myocardium 5 months after STEMI [11]. As native T1 relaxation times are strongly determined by the proportion of water content, the observed T1 decrease most likely reflects a decrease in myocardial edema, suggesting remote edema in the acute phase of MI [26]. Edema in remote myocardium is supported by previous findings of higher T2 of remote myocardium after AMI in pigs [27, 28]. Also, two studies have demonstrated a reduction in remote T2 values in patients with AMI, paralleling the changes in native T1 over time observed in our study [11, 29].

However, data on native T1 and T2 of remote myocardium in patients with AMI have been conflicting. In a study by Carberry et al. [12], remote native T1 values did not significantly change after AMI, which is in contrast to our findings and that of Bulluck et al. [11]. In the same study, they observed a significant increase in T2 values of remote myocardium over time [12], contrary to the decrease in remote T2 found by others [11, 29]. Furthermore, various studies have found a lack of difference in native T1 values [10] or T2 values [8, 9, 11] of remote myocardium compared to healthy individuals. A possible explanation for these discrepancies between studies may be that the edematous response in remote myocardium is too heterogeneous or too small to generate statistically significant differences compared to healthy individuals [30]. Moreover, differences in remote tissue responses might be due to differences in study populations in terms of AMI severity. In our study, we observed higher remote native T1 values in patients with more extensive myocardial damage, indicating that the severity of myocardial damage determines the tissue response in remote myocardium. This is in line with a previous study in which the T2 relaxation times of remote myocardium were strongly influenced by the duration of the initial ischemic insult in a porcine AMI model [28].

In addition to native T1 mapping, we also performed post-contrast T1 mapping to assess for changes in ECV of remote myocardium after AMI. ECV is a measure of the extracellular space of the myocardium and is considered a surrogate marker of fibrosis [31]. Besides fibrosis, expansion of the ECV can also occur in the presence of extracellular edema and increased cellularity. In the current study, the ECV of remote myocardium did not show a significant change over time in the total group of patients, consistent with a previous observation [12]. Edema resolution in remote myocardium would be expected to be accompanied by a reduction in remote ECV. However, the effect of edema resolution on ECV might have been counterbalanced by the effect of fibrosis formation on ECV.

In agreement with a previous study [10], remote native T1 was significantly correlated to CRP levels, independent of infarct size and MVI. In addition to that study, we also found a significant correlation between remote ECV and both CRP and fibrinogen, and a trend towards a significant correlation between remote native T1 and fibrinogen. The correlation between inflammatory markers and remote tissue characteristics suggests a link between remote edema and inflammation, which seems to be supported by previous findings from histological and imaging studies [2, 3, 32]. However, studies comparing CMR with histology in the same group of patients are needed to validate this hypothesis.

### Predictive value of remote T1 and ECV on LV remodeling

In the present study, remote native T1 was not significantly associated with changes of LV volumes at 3 months. Our results differ from the study of Carrick et al. [10], who found an association between remote native T1 and cardiac remodeling as well as MACE 6 months after AMI. This discrepancy may be partly due to the smaller sample size of our study population or the shorter follow-up period after AMI. In terms of ECV, we observed higher ECV values of remote myocardium at follow-up in patients who developed LV dilatation. This observation confirms the findings described in the study by Bulluck et al., although in that study elevated remote ECV was already found in the acute phase and remained elevated during follow-up [11]. In our study, remote ECV decreased post-AMI in patients with no LV dilatation, but remained elevated at follow-up in those who developed LV dilatation. This is in line with the study by Carberry et al., who described a correlation between expansion of remote ECV after AMI and LV dilatation at follow-up [12]. Higher ECV of remote myocardium most likely reflects early fibrotic responses, as supported by prior animal studies [33, 34]. We also found remote native T1 and ECV values to be significantly correlated with 3-month measurements of LGE infarct size. This observation may suggest that there is a relation between tissue changes in remote myocardium and the final amount of infarct fibrosis. Previous studies have shown a very close correlation between histological infarct fibrosis and LGE infarct size measured in the convalescent stage of AMI [35, 36]. However, we do not have histological data on fibrosis. It would be interesting for future studies to correlate remote native T1 and ECV with histopathology in explanted or post-mortem hearts from patients with AMI.

T1 mapping and ECV calculation provide important non-invasive insight into the pathophysiological mechanisms of cardiac remodeling after AM. However, considering the small effect sizes, the conflicting results between studies, and the unresolved technical issues in general, further studies are warranted to determine the clinical relevance of remote T1 and ECV mapping in long-term follow-up.

### Limitations

There are some limitations in this study. First, this study is limited by its relatively small sample size, and as a consequence, a lack of significance does not necessarily exclude the existence of an association. We found a statistically significant decrease in remote native T1 over time, and also higher remote ECV at follow-up in patients who developed LV dilation. Second, synthetic ECV, in which the hematocrit is not known but rather calculated from measured native T1 of blood, was used in the present study. As a result, there might be some measurement error of ECV. Nevertheless, Treibel et al. demonstrated that synthetic ECV is highly correlated to conventional ECV, with a similar relationship to histological collagen volume fraction [25]. Moreover, inflammatory markers such as selectins, interleukins and tumor necrosis factors were not determined in the present study. These markers may provide further insights into the relation between remote tissue changes and inflammation, beyond that available from our plasma CRP and fibrinogen measurements. Last, LV remodeling is a continues process and it would therefore be interesting to also have longer follow-up.

### Conclusions

Native T1 of remote myocardium decreased from baseline to 3 months in patients with reperfused first-time AMI, suggesting the resolution of remote edema. ECV of remote myocardium decreased over time in patients with no LV dilatation, but remained elevated at follow-up in those who developed LV dilatation. Findings from this study may add to an increased understanding of the pathophysiological mechanisms of adverse remodeling after AMI, which could help to define therapeutic targets for future studies to prevent adverse remodeling post-AMI.
